# Community‐Based Environmental Interventions to Prevent Alcohol Use in Adolescents: A Systematic Review

**DOI:** 10.1111/dar.70038

**Published:** 2025-09-18

**Authors:** Matheus De Carlos Oliveira, Marília Mendes‐Sousa, Luís Eduardo Soares‐Santos, Juliana Y. Valente, Sheila C. Caetano, Zila M. Sanchez

**Affiliations:** ^1^ Department of Preventive Medicine, Escola Paulista de Medicina Universidade Federal de São Paulo São Paulo Brazil; ^2^ Department of Psychiatry, Escola Paulista de Medicina Universidade Federal de São Paulo São Paulo SP Brazil

**Keywords:** adolescent, alcohol, community‐based, environmental prevent, systematic review

## Abstract

**Issues:**

This systematic review of community‐based environmental prevention strategies seeks to understand their characteristics, examine their effectiveness and identify challenges for preventing alcohol use among adolescents.

**Approach:**

A comprehensive search was conducted in MEDLINE/PubMed, Scopus and PsycINFO through December 2024. Eligible studies were randomised or quasi‐experimental designs targeting individuals under 21 years, evaluating environmental interventions (regulatory, physical or economic) with outcomes related to underage alcohol consumption and access. Two reviewers independently selected studies, extracted data and assessed risk of bias using the RoB 2 and ROBINS‐I tools.

**Key Findings:**

Regulatory strategies were the most frequent (94%; 15/16), followed by physical strategies (37%; 6/16). The most reported outcome was a reduction in alcohol availability (62%; 10/16), followed by a general reduction in consumption (37%; 6/16). Although 69% (11/16) of the studies reported positive effects, heterogeneity in study designs and terminology limited comparability. It is crucial to note that community mobilisation, although not an environmental strategy per se, was described in 81% (13/16) of the studies.

**Implications:**

The analysis indicates that the effectiveness and sustainability of environmental interventions are strongly associated with their integration with community mobilisation. This synergy, however, introduces methodological complexity, making it difficult to analyse components in isolation and to standardise evaluation.

**Conclusions:**

The findings reaffirm the value of environmental interventions, particularly regulatory ones, in preventing alcohol use among adolescents. The most promising model is multicomponent, combining actions that modify the environment with robust processes of community participation, forming an adaptable and holistic framework to promote sustainable outcomes.

**Summary**

- Regulatory measures, primarily enforcement of alcohol sales laws, were implemented in 15/16 (94%) studies; effectiveness and sustainability of environmental interventions are strongly associated with their integration with community mobilisation.
- Combined strategies (enforcement, media and community mobilisation) demonstrated higher preventive effects.
- Evidence is geographically concentrated in the United States, restricting the generalisability of effective environmental interventions.

## 1 Introduction

Adolescence is a critical developmental phase characterised by significant physical and psychological changes, often accompanied by an increased likelihood of engaging in risky behaviours [1]. Alcohol consumption during this period poses substantial risks to brain development, impairing synaptic plasticity essential for learning and memory. It also alters the structure of the prefrontal cortex, undermining decision‐making and impulse control [2]. These neurological effects can negatively influence socio‐emotional behaviours, including academic performance, mental health and social connections. Additionally, early alcohol use increases the risk of developing substance use disorders in adulthood [3, 4].

Alcohol consumption is associated with over 200 diseases and a broad spectrum of behavioural, social and health problems [5, 6]. Given these and other detrimental impacts, alcohol use represents a major global public health concern, especially among youth, who are particularly vulnerable to the effects of substance use [3, 7].

These risks underscore the urgency of preventive strategies targeting alcohol use from early life stages to avoid long‐term harm [8, 9]. Effective interventions among young people can reduce both incidence and prevalence of alcohol consumption [10, 11].

To address this issue, prevention science has advanced in implementing strategies and public policies based on understanding the factors that encourage such behaviour. Identifying environmental determinants that contribute to risky behaviours enables the development of targeted actions to mitigate these risks within the environments adolescents inhabit [12]. In this context, environmental prevention emerges as an approach grounded in the creation of systems that empower individuals and communities to recognise and manage risk factors, such as enforcing legal age limits or creating safer public environments [13]. This empowerment occurs through policies and interventions that limit exposure to unhealthy or risky behaviours while also promoting opportunities for healthier choices [14].

Although no single definition of environmental prevention exists [15, 16], it is generally understood as a set of actions and policies designed to modify contextual factors by reducing the availability and salience of behavioural triggers and opportunities. Such interventions aim, in the short term, to discourage inappropriate behaviours, such as substance use, by reducing exposure to maladaptive stimuli [17, 18, 19].

Environmental interventions can be grouped into three main categories, which often interrelate and overlap: regulatory, physical and economic [13]. Regulatory interventions involve the creation and enforcement of laws or restrictions, such as establishing the legal minimum age for alcohol consumption, prohibiting the sale of alcoholic beverages to visibly intoxicated individuals, or limiting advertising directed at such products. Physical interventions correspond to modifications in built or natural environments that influence behaviours, which may occur at the micro level, such as repositioning alcoholic beverages or unhealthy foods at points of sale, or at the macro level, through urban planning and landscaping strategies that affect access and consumption patterns. Economic interventions operate through financial mechanisms that encourage healthier choices, such as increasing taxes on alcoholic beverages or providing subsidies for nutritionally adequate foods. These initiatives underscore the role of contextual regulations in shaping behaviour, with growing attention to their application at the community level [15]. Integrating environmental prevention with community‐based strategies can strengthen these efforts, enhance policy effectiveness in local contexts and foster the social support needed to sustain change [10, 20, 21].

Community‐based interventions aim, among other goals, to shift the focus of health care from approaches centred solely on individual‐level factors to a broader perspective that emphasises transforming practices and developing policies grounded in local realities [22, 23]. In this process, the joint development of interventions with those directly affected is considered essential [24]. This approach goes beyond one‐off community mobilisation or superficial engagement, constituting an ongoing process that integrates evidence‐based academic knowledge with practical insights derived from everyday lived experiences [25, 26].

Prominent studies such as *Preventing Alcohol Trauma: A Community Trial* [27], *Communities Mobilising for Change on Alcohol (CMCA)* [28], *Project Northland* [29], the *Trelleborg Project* [10], *Sacramento Neighbourhood* [30] and *Stockholm Prevents Alcohol and Drug Problems (STAD)* [31, 32] demonstrate the potential of environmental interventions in preventing alcohol use within communities.

A relevant example was provided by Holder et al. [27], which implemented an intervention aimed at controlling the sale of alcohol to adolescents. This initiative included, in addition to reinforcing existing laws, training merchants in best practices for serving alcoholic beverages and advocacy through the media. The results demonstrated that establishments located in communities that did not receive the intervention had a 1.9 times higher probability of selling alcohol to minors compared to those in communities that benefited from the intervention [8]. While these initiatives demonstrate the potential of environmental strategies, there remains a lack of systematised knowledge on their components, effectiveness and implementation challenges across different contexts.

The growing recognition of environmental interventions highlights the importance of systematising the factors that contribute to their effectiveness. This study aims to address this gap through a systematic analysis of community‐based environmental prevention interventions targeting adolescent alcohol consumption. By providing a comprehensive review of the characteristics, modalities and outcomes of these interventions, the review aims to inform public policies and community initiatives that promote safer and healthier environments for young people. We hypothesise that interventions that integrate multiple environmental approaches are likely to be more effective in reducing adolescent alcohol consumption than those based on a single component.

## 2 Methods

This systematic review was reported in line with the Preferred Reporting Items for Systematic Reviews and Meta‐Analyses (PRISMA) checklist and the Synthesis Without Meta‐analysis (SWiM) guidelines, as proposed by Campbell et al. [33]. The protocol was prospectively registered on the PROSPERO platform (CRD42024561105) and was followed in full, with no deviations from the original plan.

### 2.1 Eligibility Criteria

This review included studies that met the following eligibility criteria: (i) preventive interventions aimed at developing or implementing strategies or policies in physical, economic and/or regulatory environments; (ii) approaches exclusively targeting the prevention of alcohol use among individuals under the age of 21; and (iii) studies reporting outcomes related to alcohol consumption, including lifetime use, recent use and binge drinking, as well as access to alcoholic beverages through direct purchase, family members, friends or other community members.

Additionally, the following were excluded: (i) studies whose interventions were limited to school or family settings without including the broader community context; (ii) studies that did not specify the adolescent age range of the target population; (iii) protocols, literature reviews or policy analyses lacking empirical field data related to alcohol prevention; and (iv) observational studies, as they do not provide comparisons between intervention and control groups. The inclusion of randomised controlled trials and quasi‐experimental studies was prioritised due to their greater ability to provide robust and reliable estimates of intervention effects, thereby reducing bias compared to observational studies, which are often considered as the ‘other standard’ [34, 35]. Conditions of comparison typically included communities or regions without active implementation of environmental prevention interventions, or those implementing alternative interventions such as purely educational or individual‐focused strategies.

These inclusion and exclusion criteria were established to ensure consistency and relevance among the studies reviewed, focusing on interventions with specific approaches and measurable outcomes in environmental alcohol prevention for adolescents. Although many prevention strategies incorporate environmental components, only studies implementing interventions within regulatory, physical or economic categories were included [19].

### 2.2 Information Sources and Search Strategy

The research question was structured using the PICO model (Population, Intervention, Comparison, Outcome) guided our search strategy to ensure a comprehensive approach to the research question to define the scope of the review and inform the search strategy [36]. The population included adolescents and young adults (12–21 years old), addressing underage drinking in countries where the legal drinking age is 21. Interventions focused on environmental approaches to prevent alcohol use, with comparisons made to settings that lacked such interventions. The primary outcomes assessed were reductions in alcohol consumption among adolescents.

The search was conducted in March 2024, including articles published up to that date. Systematic searches were performed in the MEDLINE/PubMed and Scopus databases using the search terms: ‘adolescent AND prevention AND (community OR environmental) AND alcohol’. In PsycINFO, the search used: ‘adolescent AND prevention AND (community) OR (environmental) AND alcohol’. While no restrictions were placed on publication dates, the search was limited to articles published in English. Initial tests with more complex search strings showed minimal improvement in the retrieval of relevant articles. Thus, simpler but highly sensitive search strings were selected to ensure comprehensiveness.

Near the completion of the article, an update of the literature search was conducted. Thus, at the end of 2024, new searches were performed in the same databases using the previously defined search strategies. However, between March and December 2024, no new studies meeting the inclusion criteria of this review were identified.

### 2.3 Selection Process

Following systematic review guidelines, two independent reviewers, blind to each other's initial decisions, conducted the article selection using the Rayyan tool [37]. Any conflicts were resolved by a third reviewer, ensuring consistency and transparency. This rigorous process enhanced the reliability of the selected articles and minimised potential biases [38].

### 2.4 Data Collection Process

The data regarding the characteristics of the selected studies were extracted by the two independent reviewers using a tool specifically developed for this purpose. The information collected included the study reference, methodological design, sample characteristics, intervention components and the main outcomes assessed.

### 2.5 Study Risk of Bias Assessment

The methodological quality of the included studies was conducted independently by two reviewers, based on the principles established by Cochrane. For randomised controlled trials, the Risk of Bias 2 (RoB 2) tool was applied, while for quasi‐experimental studies, the risk of bias in non‐randomised studies of interventions (ROBINS‐I) tool was used. This approach enabled the identification of potential sources of bias and the evaluation of the robustness of the evidence presented [39, 40, 41].

The RoB 2 tool assesses bias across five mandatory, non‐modifiable domains. Within each domain, signalling questions guide a judgement of ‘Low risk of bias’, ‘Some concerns’ or ‘High risk of bias’. An overall risk of bias judgement is then assigned to each outcome [40]. The ROBINS‐I tool is structured into seven domains that encompass different stages of a study. Each domain is assessed through signalling questions that guide reviewers in assigning a judgement across five levels: low, moderate, serious, critical or no information [41].

To support the visual presentation of risk of bias assessments, the online tool ROBVIS (Risk Of Bias VISualization) was employed [42]. Importantly, individual risk of bias ratings were not used as sole criteria for study inclusion or exclusion in the review; instead, they served as complementary elements in the critical appraisal of the methodological quality of the evidence.

### 2.6 Synthesis Methods

The review synthesised findings by categorising studies into three primary types of environmental interventions: regulatory, physical and economic. To explore whether differences in study characteristics influenced the effects of the interventions, exploratory analyses were conducted and the results are presented in Table 1. Each study was examined in terms of the type of intervention, target population, outcome measures and methodological design, aiming to identify both commonalities and distinctions among the studies analysed. The synthesis emphasised effectiveness in reducing alcohol consumption and limiting access to alcohol, employing descriptive statistics where applicable. Due to the high heterogeneity in intervention designs, outcome measures and implementation contexts, a robust meta‐analysis was not feasible. Therefore, an outcome evaluation approach was adopted based on the results reported by the included studies, taking into account various forms of effect presentation, such as statistical significance levels (*p*‐values), mean differences and effect size estimates when available. In addition, a narrative synthesis was employed to preserve the specificity of the findings and to reflect the heterogeneity of the analytical strategies used across studies for each outcome analysed.

**TABLE 1:** Studies Implementing Environmental or Community Strategies for Preventing Alcohol Use Among Adolescents (*n* = 16).

Reference and country	Study design	Sample	Intervention components	Category of environmental actions	Outcome
Wagenaar et al. [28], USA	RCT	*n* = 15 communities	Social marketing; Community mobilisation; Sales control; Regulation monitoring and enforcement.	Regulatory.	Greatest effect on ‘Alcohol retailers: On‐ premise consumption’ (d = 1.18, *p* = 0.04). Substantial effects on ‘People aged 18–20’ (d = 0.76, *p* = 0.01). No effect for ‘High school seniors’ and ‘Alcohol retailers: Off‐premise sales’.
Wallin & Andreásson [43], Sweden	RCT	*n* = 164 licensed establishments	Training of staff in responsible beverage service; Policy initiatives for purchase attempts by adults appearing underage; Enforcement of alcohol regulations.	Regulatory.	No differences between intervention and control areas
Rehnman, Larsson, & Andréasson [44], Sweden	Quasi‐ experimental	*n* = 55 grocery stores	Steering group and parent meetings; Postcards to parents of adolescents; Merchant meetings; Store visits; Parental store monitoring; Letters to storekeepers; Sales staff training; Media advocacy.	Regulatory; Physical.	No difference between intervention and comparison areas, partly due to contamination effect in the comparison area where similar activities were conducted by the local community
Wagenaar, Toomey & Erickson [45], USA	Quasi‐ experimental	*n* = 20 cities	Retail management training for alcohol establishments and compliance checks.	Regulatory.	**↓**17% (RR = −0.17, *p* = 0.05) in the likelihood of sales immediately following a compliance check
Wagenaar et al. [46], USA	Quasi‐ experimental	*n* = 50 states	Media coverage; State alcohol policy.	Regulatory.	Magnitude of differences associated with RUD coalitions: effect size of 1.10 in media coverage, 0.46 in enacted state policies, −0.44 in youth alcohol consumption behaviours, and −0.16 in alcohol‐related driving and fatal driving accidents.
Treno et al. [30], USA	Quasi‐ experimental	*n* = 280 Sacramento blocks	Community awareness and mobilisation; Responsible beverage service; Underage access enforcement; Intoxicated patron enforcement.	Regulatory.	**↓**in police‐reported approaches, and in aggregate EMS outcomes, approaches and motor vehicle accidents.
Moore et al. [47], USA	Quasi‐ experimental	*n* = 51 purchase attempts in stores	Social marketing; Community mobilisation; Responsible service training.	Regulatory.	38% of stores did not request ID in the first reward and reminder round, but 0% did not request ID in the subsequent two rounds.
Flewelling et al. [48], USA	RCT	*n* = 36 communities	‘Reward and reminder’ program for clerks and retailers; Increased enforcement of retail sales laws; Increased enforcement of underage alcohol consumption laws; Efforts to reduce and prevent underage drinking at parties; Strategic media advocacy.	Regulatory; Physical.	Effects only for the underage sales.
Kraus et al. [49], Germany	Quasi‐ experimental	*n* = 8 communities	Coordination and structure; Communication and public relations; Youth protection; Activities for youths; Activities for parents.	Physical.	Few differences between HIG and LIG in terms of alcohol consumption and negative consequences.
Schelleman‐Offermans, Knibbe & Kuntsche [50], Netherlands	Quasi‐ experimental	*n* = 2 communities	Active mobilisation of parents and teachers; Sales control, restricting alcohol supply by retailers, with stricter policies for age verification; Alcohol sales restrictions at certain times or areas; Punitive measures for retailers selling alcohol to minors.	Regulatory; Physical.	**↓**15% risk of progression to drunkenness for weekly drinking adolescents, but no reduction in the risk of weekly drinking among adolescents in the intervention region; No intervention effects on intermediate goals among 14–15‐year‐olds; Positive effect on alcohol supply by parents and specific alcohol rules among 13‐year‐olds.
Jansen et al. [51], Netherlands	Quasi‐ experimental	*n* = 8800 adolescents	Social marketing through media campaigns; Community mobilisation involving parents and other significant adults in youth environments; Sales control; Monitoring and regulation of alcohol sales; Regulation monitoring and enforcement.	Regulatory; Physical.	**↓**recent alcohol consumption and binge drinking, after 1 year with effects maintained after 5 years, particularly among second‐year high school students.
Komro et al. [52], USA	RCT	*n* = 7 communities	Social marketing; Community mobilisation; Sales control; CONNECT conducted universal screening and brief interventions in schools, with individual motivation sessions.	Regulatory; Physical.	**↓**past 30‐day alcohol use and binge drinking in students exposed to CMCA, CONNECT, or both, with varying effect magnitudes over the 2.5‐year intervention period.
Wagenaar et al. [9], USA	RCT	*n* = 5 communities	Community mobilisation; Sales control.	Regulatory.	Alcohol acquisition by youth from various sources (parents, adults over 21, peers under 21 and stores).
Van Ryzin, Lee & Biglan [53], USA	RCT	*n* = 8 communities	Community mobilisation; Reward and reminder.	Regulatory.	Significant results for intervention points of sale: Requested ID (*p* < 0.05) and Willing to Sell (*p* < 0.05); Asked about Age: not significant.
Duch et al. [21], Spain	Quasi‐ experimental	*n* = 9 supermarket chains	Trainer and seller training; Community mobilisation; Media campaign.	Regulatory.	Supermarkets that completed the training: **↓**average alcohol sales from 76.9% in 2018 to 45.5% in 2020; **↑**age verification from 3.8% to 45.4%; **↑** proof of age request from 15.4% to 72.7%.
Saltz, Paschall & O'Hara [54], USA	RCT	*n* = 24 cities	Community mobilisation; Social marketing; Sales control.	Regulatory.	**↓**17% in alcohol‐related crashes among drivers aged 15–30, equivalent to 310 less accidents.

Abbreviations: **↓**: decrease/reduction, **↑**: increase/rise; AUDIT‐C, Alcohol Use Disorders Identification Test—Consumption; CMCA, Communities Mobilising for Change on Alcohol; CONNECT, a school‐based universal screening and brief intervention; EMS, emergency medical services; HIG, high intervention fidelity group; ID, identification; LIG, low intervention fidelity group; RCT, randomised controlled trial; RUD, reducing under‐age drinking; SUD: supply for unsupervised drinking.

## 3 Results

**FIGURE 1:**
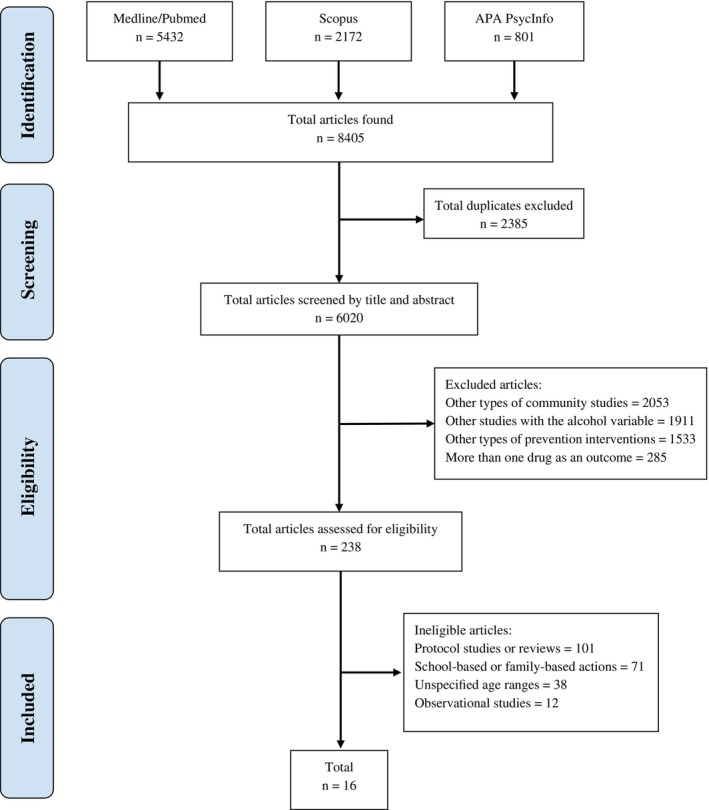
PRISMA Flow Diagram.

The PRISMA flow diagram [55] detailing the article selection process is presented in Figure 1. Initially, 8405 articles were identified: 5432 from MEDLINE/PubMed, 2172 from Scopus and 801 from APA PsycInfo. During the screening process, 2385 articles were excluded as duplicates, leaving 6020 for title and abstract review. Of these, 5782 were excluded for failing to meet the eligibility criteria, resulting in 238 articles for further consideration. After the final analysis, 16 articles were selected for inclusion in this review based on the exclusion criteria.

### 3.1 Study Characteristics

Table 1 presents the 16 studies selected for this review, of which nine employed a quasi‐experimental design and seven used randomised methods. A temporal analysis of the studies reveals a modest yet noticeable increase in scientific output focused on the prevention of alcohol use through environmental interventions at the community level, particularly targeting adolescents. This growth is especially evident over the past decade: between 2000 and 2010, six studies were published; between 2011 and 2020, this number rose to nine; and from 2021 to the final date of data extraction, one additional study was incorporated into the literature. This progression represents an approximate 30% increase in the volume of publications over time.

During the analysis of the included studies, we observed a recurring use of specific terms to describe environmental prevention actions. Although these terms were not used in the initial search strategy, their identification may inform future refinements to systematic search approaches. Commonly recurring terms include ‘sales control’, ‘access regulation’, ‘enforcement of prevention laws’, ‘access control’, ‘monitoring alcohol supply’, ‘environmental policies’, ‘physical environment’, ‘prevention policies’ or simply ‘policies’, the latter being a more generic way to refer to public policies aimed at preventing the availability or sale of alcohol. Although these terms were not used in the initial search strategy, their identification may inform future refinements to systematic search approaches. These recurring terms were identified during full‐text review and may guide the refinement of terminology in future search strategies and prevention classifications. Regarding geographical distribution, the United States leads the production of studies, contributing 10 of the 16 articles. European countries, such as the Netherlands and Sweden, each contributed with two studies, while Germany and Spain each published one study. Notably, no studies from Latin America, Africa or Asia were identified.

The characteristics of the samples in the included studies appeared to be relatively homogeneous, as all were focused on adolescents, an essential criterion for inclusion in this review. However, some age variation was observed across the studies, with some targeting younger adolescents (ages 12 to 16) and others focusing on older age groups within adolescence, ranging from 17 to 21 years old.

The studies varied from medium (approximately 1 year) to long (up to a decade). It is noteworthy that the implementation of interventions did not always align with the beginning of the research, as preparatory steps, such as needs assessment and stakeholder mobilisation, were often completed before the actions were executed. The deployment of interventions also varied based on factors like the targeted level of prevention, the specific goals of each study and the timeframe available for implementation.

### 3.2 Categories of Environmental Actions Implemented

Among the three conceptual categories adopted for environmental prevention—regulatory, physical and economic—the 16 analysed studies demonstrated a limited diversity of interventions implemented in each study. Of the reviewed studies, 10 focused solely on regulatory actions, five combined both physical and regulatory measures and one implemented a physical intervention. No study included interventions focused on economic aspects of environmental prevention aimed at reducing adolescent alcohol consumption (see Table 1).

Regulatory interventions aimed at preventing adolescent alcohol consumption were the most employed, implemented in 94% (*n* = 15) of the reviewed studies. This category emerged as the most widely used within environmental prevention efforts. Key measures emphasised the enforcement of existing laws, often referred to as ‘Minor Access Law Enforcement’ or ‘Sales Control’. A typical example involved inspections to ensure compliance with alcohol sales regulations in commercial establishments such as bars, markets and restaurants, a practice known as ‘Compliance Monitoring’. The approaches to monitoring compliance varied across studies: some relied on law enforcement agencies for inspections, while others engaged the community in supporting these actions or conducting independent investigations, often referred to as ‘Reward and Reminder’ programs.

Among the reviewed studies, 37% (*n* = 6) reported interventions targeting modifications to the physical environment. These measures included actions designed to reduce physical factors that promote alcohol‐related behaviours. One common approach involved implementing distancing rules between alcohol‐selling establishments, such as bars and locations frequented by adolescents, such as schools and parks. Additionally, some studies implemented strategies to restrict the availability of alcoholic beverages within communities, including limiting the density of alcohol‐selling establishments and designating specific zones for alcohol consumption.

### 3.3 Community Mobilisation and Media Use

All reviewed studies incorporated environmental interventions and integrated strategies for community mobilisation, often paired with mass media campaigns. Community mobilisation was a prominent feature, present in 81% (*n* = 13) of the interventions, and emerged as a critical component in preventing alcohol consumption among adolescents. Although not categorised as an environmental strategy, community mobilisation frequently complemented such approaches in most studies. Typical mobilisation activities included engaging policymakers, community leaders, parents, alcohol retailers and adolescents (see Table 1).

Interventions that included community mobilisation as a supporting element for planning and implementation proved significantly more effective in prevention. The enactment or reinforcement of laws aimed at preventing alcohol use among minors was often coupled with mass media campaigns and advocacy initiatives. These combined strategies sought to bolster public support for environmental interventions, amplify the effects of regulatory policies and enhance compliance with alcohol prevention laws.

### 3.4 Outcomes Investigated

This review identified two main outcomes most frequently referenced in the analysed studies: (i) a reduction in alcohol consumption among adolescents, reported in six articles (37%); and (ii) decreased access to alcohol, achieved through measures such as location‐based sales restrictions, reduced availability, or responsible service practices, observed in 10 studies (62%).

### 3.5 Effectiveness by Outcome

Of the 16 analysed articles, 11 (69%) demonstrated significant effectiveness in preventing adolescent alcohol use compared to control groups. Due to the specificity of intervention designs and the varied approaches, aggregating results into a single statistical summary was not feasible. Instead, an individual outcomes analysis was conducted (see Table 1). Among the six studies targeting reductions in alcohol consumption, four reported significant effectiveness. Of the 10 studies focused on limiting access to alcohol, seven showed significant effects relative to control groups.

### 3.6 Risk of Bias

**FIGURE 2:**
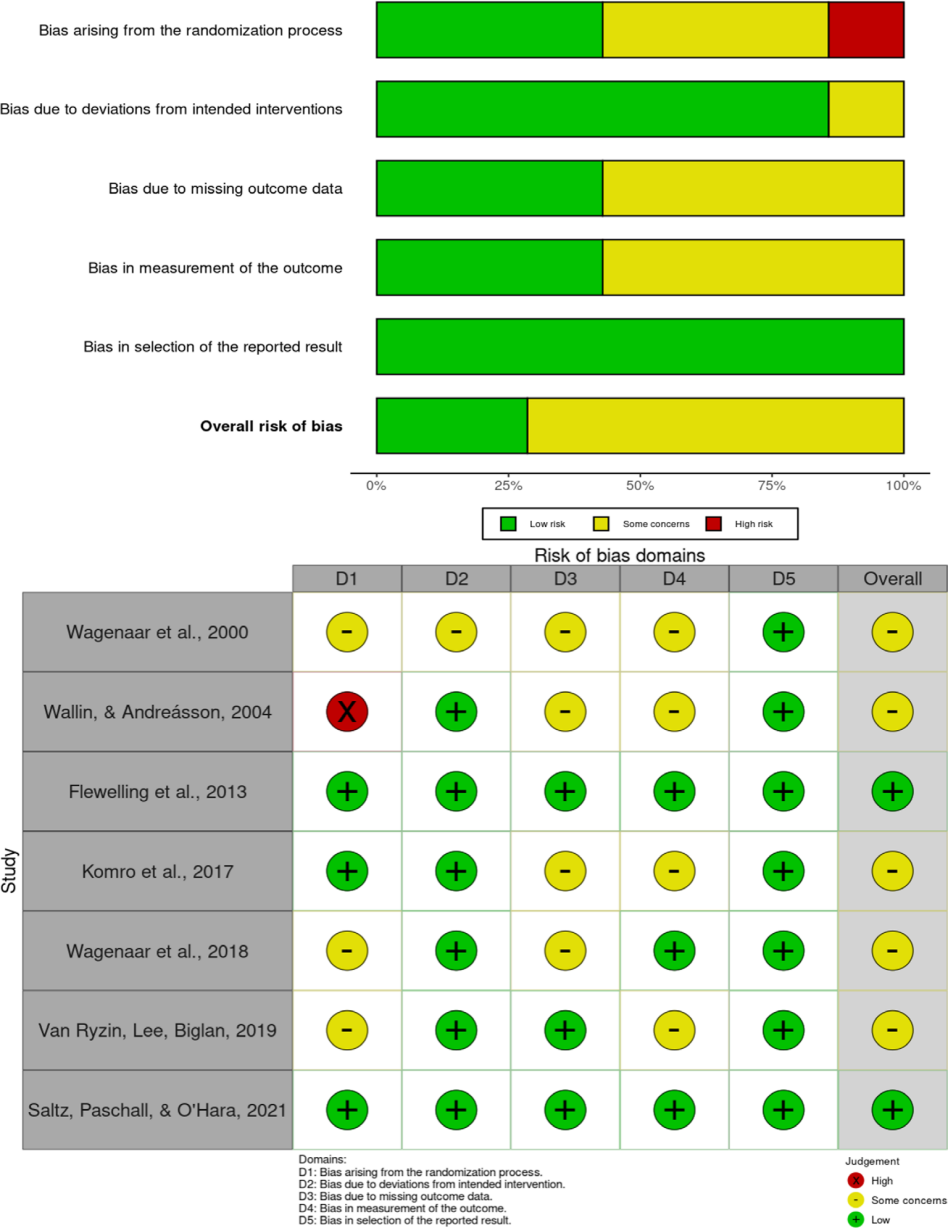
Summary and Risk of Bias Ratings of Randomised Trials (*n* = 7).

For the randomised studies, the risk of bias assessment was conducted using the Cochrane RoB 2 tool (see Figure 2). Among the seven articles evaluated, only two were rated as having a low risk of bias across all assessed domains: the studies by Flewelling et al. [48] and Saltz, Paschall and O'Hara [54], which demonstrated robust methodological designs with no identified shortcomings. The remaining five studies were rated as having some concerns regarding risk of bias. Specifically, Wallin and Andreásson [43] showed a high risk of bias in the domain related to the randomisation process, although their overall risk rating remained as ‘some concerns’. Wagenaar et al. [28], on the other hand, were classified as having some concerns specifically in terms of adherence to the intended intervention, in contrast to the other studies, which were considered low risk in this domain. It is important to highlight that, in all included randomised studies, the risk of bias regarding the selection of reported outcomes was assessed as low, which contributes to the overall confidence in the findings.

**FIGURE 3:**
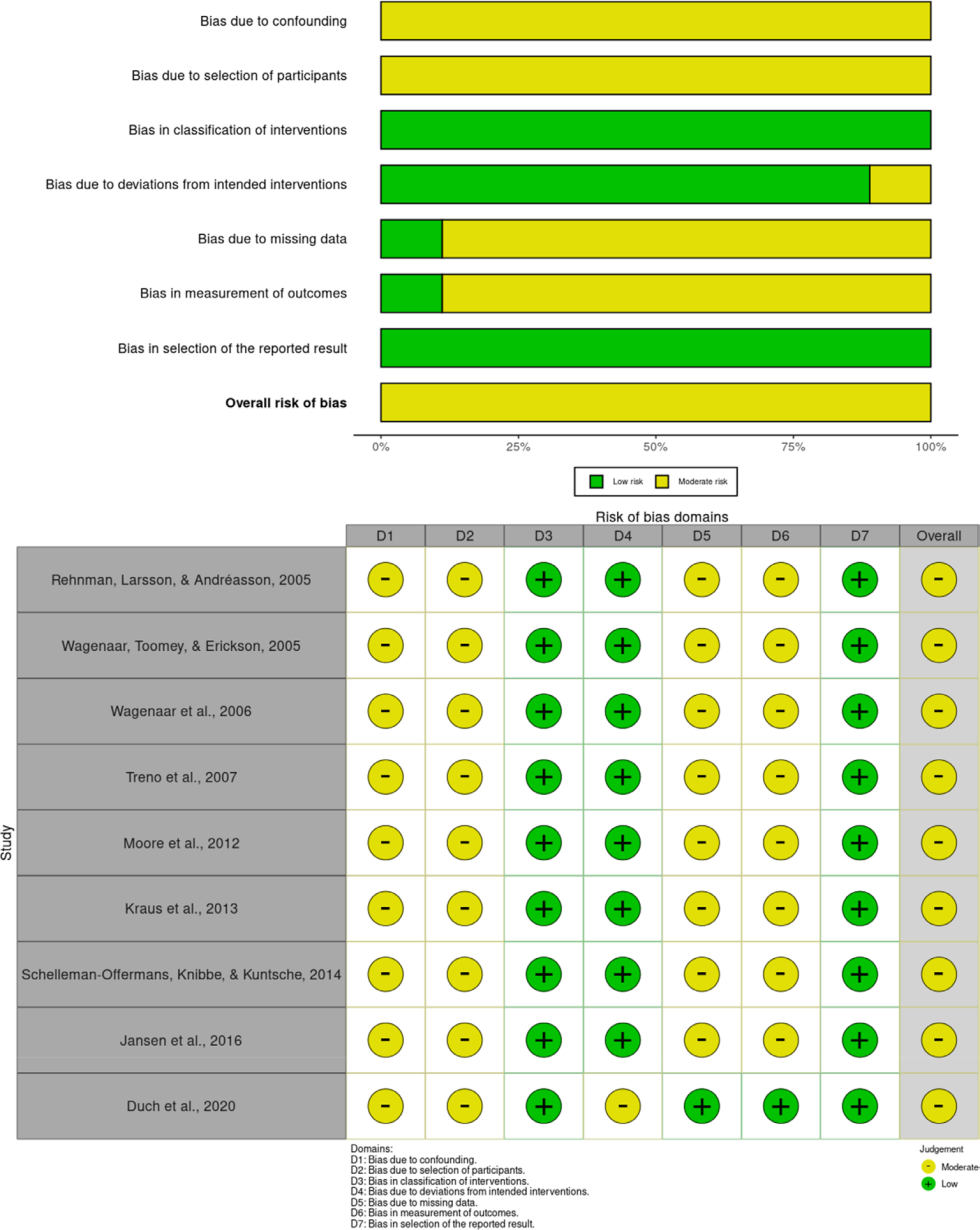
Summary and Risk of Bias Ratings of Non‐Randomised Trials (*n* = 9).

For the nine non‐randomised studies, the ROBINS‐I tool was applied, revealing a moderate risk of bias across all articles (see Figure 3). None of these studies were rated with a high risk of bias for any criterion, and each reported relevant information adequately. Within ROBINS‐I's assessment factors, classification of the intervention, deviations from intended interventions and selection of reported outcomes generally presented a low risk of bias. However, confounding factors and participant selection bias were classified as presenting a moderate risk (see Figure 3).

## 4 Discussion

This review synthesised findings from 16 studies that evaluated the effectiveness and implementation of Environmental Interventions. A diverse range of terms was used to describe these interventions, with a consistent emphasis on the community as the central unit of analysis. Notably, most studies that produced significant outcomes were conducted in the United States. Access control measures emerged as the most commonly employed strategy. Importantly, the studies reporting the most favourable results often combined these measures with community mobilisation efforts, underscoring the synergistic potential of integrating these approaches.

Almost all the articles included actions aimed at establishing regulatory measures to restrict adolescent access to alcohol, making this the most prevalent intervention. The emphasis of the review on adolescents and young adults may explain the predominance of studies implementing regulatory environmental interventions, a finding supported by the literature highlighting the high potential effectiveness of such measures in preventing alcohol use within this population [8, 14, 27]. However, this prioritisation may lead to the underrepresentation of other environmental prevention strategies, such as physical or economic measures.

The consolidation of our findings was challenged by significant terminological inconsistency. This review underscores the inconsistent terminology used to describe environmental interventions, particularly those involving community mobilisation. Many studies favour terms such as ‘community’ rather than ‘environmental prevention’, often failing to distinguish between levels of prevention. This lack of clarity may stem from the frequent use of ‘community’ as the primary unit of analysis, creating conceptual ambiguity [13, 15]. Consequently, the overlap between community factors and environmental actions may foster the misconception that these terms are interchangeable or inherently connected [16]. The concept of environmental prevention, as outlined by Oncioiu et al. [14], demonstrates that physical, economic or regulatory practices can be implemented independently of a community‐based approach. Likewise, community interventions such as Communities That Care and PROSPER can be designed without necessarily incorporating practices derived from environmental prevention [19, 56].

As a direct consequence of this conceptual ambiguity, establishing a standardised framework to assess the effectiveness of these interventions becomes difficult [16, 27]. Beyond conceptual challenges, methodological limitations, such as small sample sizes, short follow‐up periods and insufficient rigour in control conditions, further compromise the identification of the most effective actions and components. In this regard, a clear definition of methodological principles guiding environmental interventions is essential to align theory with objectives, thereby fostering more consistent results in preventing adolescent alcohol use [12].

Equally critical, yet frequently neglected, are contextual moderators of effectiveness, whose relevance may vary depending on the study focus. Among them, socioeconomic status, differences between urban and rural contexts and the characteristics of political environments stand out. These variables influence not only the feasibility of implementation but also the outcomes achieved and have been recognised by Burkhart et al. [16] as central elements in discussions on the dynamics of environmental prevention.

In light of these challenges, the findings point to the superiority of multicomponent approaches. A notable finding is the diversity of individual factors that each study needed to address. Strategies implemented ranged from restrictions on alcohol sales and age verification to advertising regulation, targeted policing, conditional licensing and the imposition of penalties. Some initiatives even combined environmental measures with efforts aimed at influencing family norms and parenting styles [57, 58]. From this perspective, environmental interventions, when integrated with other levels of prevention, such as universal, indicated or selective, can offer multicomponent approaches [18], providing greater potential for effectiveness and sustainability compared to interventions focusing solely on individual‐level aspects [59, 60].

The central and integrative element of these multicomponent strategies has proven to be community mobilisation. The strengthening of policies to enforce stricter controls on adolescent alcohol consumption is generally combined with such mobilisation, a key component in ensuring adherence and engagement. A central finding of this review indicates that the integration of environmental actions with community mobilisation significantly enhances outcomes in preventing adolescent alcohol use. Community mobilisation contributes to the development and implementation of interventions by enabling strategies to be adapted to local contexts and by overcoming specific barriers [17, 23, 61]. Our findings echo challenges identified by Segrott and colleagues [62], who stress that the future of prevention science depends on strengthening long‐term partnerships with communities, balancing participatory approaches with evidence‐based principles, and developing clear frameworks to assess the quality and impact of community engagement.

This integration is well illustrated by established implementation models. Examples of approaches that combine community support with systematic practices grounded in scientific principles include *Communities That Care (CTC)* [63, 64], *Community‐Based Prevention Marketing (CBPM)* [65, 66], *Project Northland* [29, 67] and *Communities Mobilising for Change on Alcohol (CMCA)* [9, 28]. These models illustrate how the articulation between community engagement and regulatory strategies can enhance the effectiveness of interventions aimed at preventing adolescent alcohol use.

It is crucial, however, to clearly define what truly constitutes mobilisation. For an intervention to be considered community‐based, central aspects concerning the practical participation of individuals affected by the intervention must be incorporated into the working process, both consultative and practical [24, 26]. Although community mobilisation is often seen as a practical component, in many studies it extended beyond operational support, aiming to foster sustainable perspectives for new policies and to increase the likelihood that such measures would be adopted as public policy after the conclusion of studies [9, 14, 68]. In some cases, there may be a misinterpretation of the defining attributes of community‐based interventions, with mobilisation being regarded as sufficient for this feature to be considered prominent.

Looking ahead, parallels can be drawn between the historical trajectory of environmental prevention and contemporary demands in the field. This trajectory should not be understood merely as a linear evolutionary process but as the consolidation of the foundations underpinning prevention and implementation science [69]. From the first study included in this review, published in 2000, to the most recent in 2021, more than two decades have been marked by consistent advances that strengthened the effectiveness of community‐based environmental prevention interventions.

A striking example of this evolution is the increasingly dynamic role of media integrated into community interventions [61]. While early studies already identified media as a promising resource for expanding the reach of policies and strengthening actions in environmental contexts, the advent of the internet and the widespread diffusion of digital platforms significantly amplified this potential [21, 70]. Today, these tools are systematically incorporated into prevention strategies [11], not so much as spaces for the direct implementation of environmental actions, but rather as strategic channels to support, influence and reinforce community interventions [54, 68, 71].

However, the evolution of the field has been geographically uneven. Over the past two decades, the United States has dominated research on environmental prevention interventions targeting adolescent alcohol use, driving a substantial increase in publications. Yet geographical diversity remains limited: while some studies were conducted in Europe, regions such as Latin America, Asia and Africa remain underrepresented. This imbalance likely reflects shared characteristics among countries where such interventions have been implemented, including greater economic resources, consolidated histories of prevention policies focused on adolescents and robust academic research traditions on the subject. The predominance of studies from high‐income countries restricts the generalisability of findings and reflects the more favourable conditions for adopting and sustaining such policies in these contexts [72, 73]. In Africa, for example, alcohol‐related harms are severe, but research remains fragmented and underfunded, prompting calls for a continent‐wide alliance to strengthen collaboration, capacity building and evidence generation in the region [74].

To overcome this limitation and move forward, it is imperative to include economic evaluations assessing the scalability and adaptability of these approaches across different political contexts, reinforcing the importance of interventions that integrate implementation science frameworks and process evaluations alongside outcome measurement. This aspect becomes even more relevant given the scarcity of robust evidence in the reviewed studies regarding the long‐term sustainability and maintenance of interventions, dimensions fundamental to ensuring that community‐based environmental strategies are effectively incorporated and sustained as public policies.

Such expansion is particularly urgent, as alcohol control policies gain broader international acceptance, enabling more countries to implement comprehensive prevention strategies [75], thereby expanding the geographical scope of documented experiences and incorporating diverse cultural, regulatory and structural contexts into adolescent alcohol prevention research.

It is important to acknowledge, however, the inherent limitations of this review. A significant limitation of this systematic review is the exclusion of observational studies in favour of experimental and quasi‐experimental designs. While this choice aimed to reduce the risk of bias and ensure greater internal validity, it may have inadvertently omitted insights from real‐world interventions, especially in settings where trial designs are less feasible. A further limitation is that our search strategy may have overlooked relevant studies addressing environmental interventions in less restrictive ways, such as nudges, changes in physical environments or outlet density regulations, particularly when these did not explicitly use prevention‐related terminology in their keywords.

Moreover, interventions conducted in university settings were excluded, as the inclusion criteria restricted the age range 12–21 years. Many of these studies did not specify participants' ages, treating university populations as homogeneous groups and thus overlooking those who were still under the legal drinking age. In summary, the findings of this review reaffirm the crucial role that environmental prevention interventions play in addressing adolescent alcohol consumption, particularly when grounded in community‐based approaches. These actions, whether regulatory, physical or economic, demonstrate substantial potential to shape behaviour and reduce alcohol use, especially when implemented through multicomponent models. Moreover, when combined with community mobilisation, such approaches provide a holistic and adaptable framework that engages stakeholders and fosters sustainable outcomes.

## Author Contributions

**Matheus De Carlos Oliveira:** data collection, data extraction, drafting of the manuscript. **Marília Mendes‐Sousa:** data collection, data extraction, drafting of the manuscript. **Luís Eduardo Soares‐Santos:** data collection, data extraction, drafting of the manuscript. **Juliana Y. Valente:** writing – review and editing. **Sheila C. Caetano:** writing – review and editing. **Zila M. Sanchez:** conceptualization, supervision, writing – review and editing.

## Conflicts of Interest

The authors declare no conflicts of interest.

## Data Availability

Data sharing not applicable to this article as no datasets were generated or analysed during the current study.
